# Analysis of Wholesale Cigarette Sales in Canada After Menthol Cigarette Bans

**DOI:** 10.1001/jamanetworkopen.2021.33673

**Published:** 2021-11-09

**Authors:** Michael Chaiton, Robert Schwartz, Anasua Kundu, Christopher Houston, Robert Nugent

**Affiliations:** 1 Ontario Tobacco Research Unit, Toronto, Ontario, Canada; 2 Dalla Lana School of Public Health, University of Toronto, Toronto, Ontario, Canada; 3 Office of Research and Surveillance, Tobacco Control Directorate, Health Canada, Ottawa, Ontario, Canada

## Abstract

This economic evaluation uses wholesale cigarette sales data from manufacturers to compare cigarette sales before and after implementation of provincial and federal bans of menthol cigarettes in Canada.

## Introduction

Research on the implications of menthol cigarette bans is crucial to inform evidence-based decision-making and policy changes.^1,2^ In Canada, a series of provincial bans were implemented starting in May 2015, until a federal ban took effect in October 2017. To evaluate the bans, we assessed the overall change in cigarette sales associated with the implementation of these menthol cigarette bans across all provinces in Canada between 2010 and 2018.

## Methods

We used wholesale cigarette sales data that were reported to Health Canada by manufacturers, which are required to report by province, each brand of tobacco product, the number of units sold, package sizes, and the value of the units sold pursuant to the Tobacco Reporting Regulations (SOR/2000-273) enacted under the Tobacco and Vaping Products Act. Cigarette sales are reported monthly, and returns to companies from wholesalers and retailers are reported as negative values. All data are subject to future review because of resubmissions by companies and audits by Health Canada. The University of Toronto Research Ethics Board deemed this study exempt from review because it did not use data on human subjects. We followed the Consolidated Health Economic Evaluation Reporting Standards (CHEERS) reporting guideline for model-based economic evaluation.^3^

For each month, net unit cigarette sales for each province were calculated for the period between October 1, 2010, and December 31, 2018, and the data were analyzed from January to May 2021. For comparisons of sales in the provinces (adjusted for the fixed effects of province) and to control for seasonality, the wholesale cigarette sales were converted to a measure representing the percentage change in sales from the same month in the previous year in that province. An indicator was created to represent the presence of a menthol cigarette ban during the month when the first set of regulations was implemented for that jurisdiction (provincial or national ban).^1^

The interrupted time series regression analyses were performed using Stata 14, version 14.2 (StataCorp, LLC), and the Durbin-Watson statistic and Prais-Winsten regression were used to account for autoregression. The regression models also included a trend indicator representing the difference in slope after the bans.^4^ The regression models were run using the Cochrane-Orcutt transformation with a search for e (representing error) performed for the value of ρ that minimized the sum-of-squared errors of the transformed equation.^5^ Clustering by province was also specified. Separate analyses were then run by province without the trend indicator to assess only the magnitude of the change associated with the ban. The threshold for statistical significance was *P* = .05, and the tests were 2-tailed.

## Results

Menthol cigarette sales increased gradually in all 10 Canadian provinces from 2013 until the menthol cigarette ban was implemented. After the bans, sales of menthol cigarettes decreased to 0 in all provinces, and the overall percentage change in cigarette sales for the same month in the previous year was 4.6% (Table and Figure). The decrease to 0 menthol cigarette sales across the country suggests compliance with the ban within legal sales channels.

**Table.** Interrupted Time Series Regression Results for Menthol Cigarette Bans in Canada

	Date of menthol ban	Model-based change in sales associated with the ban, % (95% CI)^a^	*P* value
Canada overall trend model			
Ban implementation	Province specific: May 2015-October 2017	−4.6 (−8.2 to −1.0)	.02
Trend before the ban		0.001 (−0.002 to 0.004)	.48
Trend after the ban		−0.06 (−0.21 to 0.09)	.39
Provincial model (ban effect size only)			
Nova Scotia	May 2015	−1.6 (−7.5 to 4.2)	.59
Alberta	October 2015	−8.4 (−13.5 to −3.4)	.001
New Brunswick	January 2016	−7.6 (−13.7 to −1.5)	.02
Ontario	January 2017	−6.3 (−14.6 to 2.0)	.13
Quebec	August 2016	−1.7 (−6.2 to 2.7)	.44
Newfoundland and Labrador	October 2017	−9.5 (−19.4 to 0.4)	.06
Prince Edward Island	October 2017	−5.1 (−22.5 to 12.4)	.57
Saskatchewan	October 2017	−14.0 (−21.9 to −6.1)	.001
Manitoba	October 2017	−7.1 (−14.5 to 0.22)	.06
British Columbia	October 2017	−1.1 (−8.3 to 6.2)	.77

^a^ Percentage change from sales in the same month in the previous year for each province for overall cigarette sales.

**Figure.**
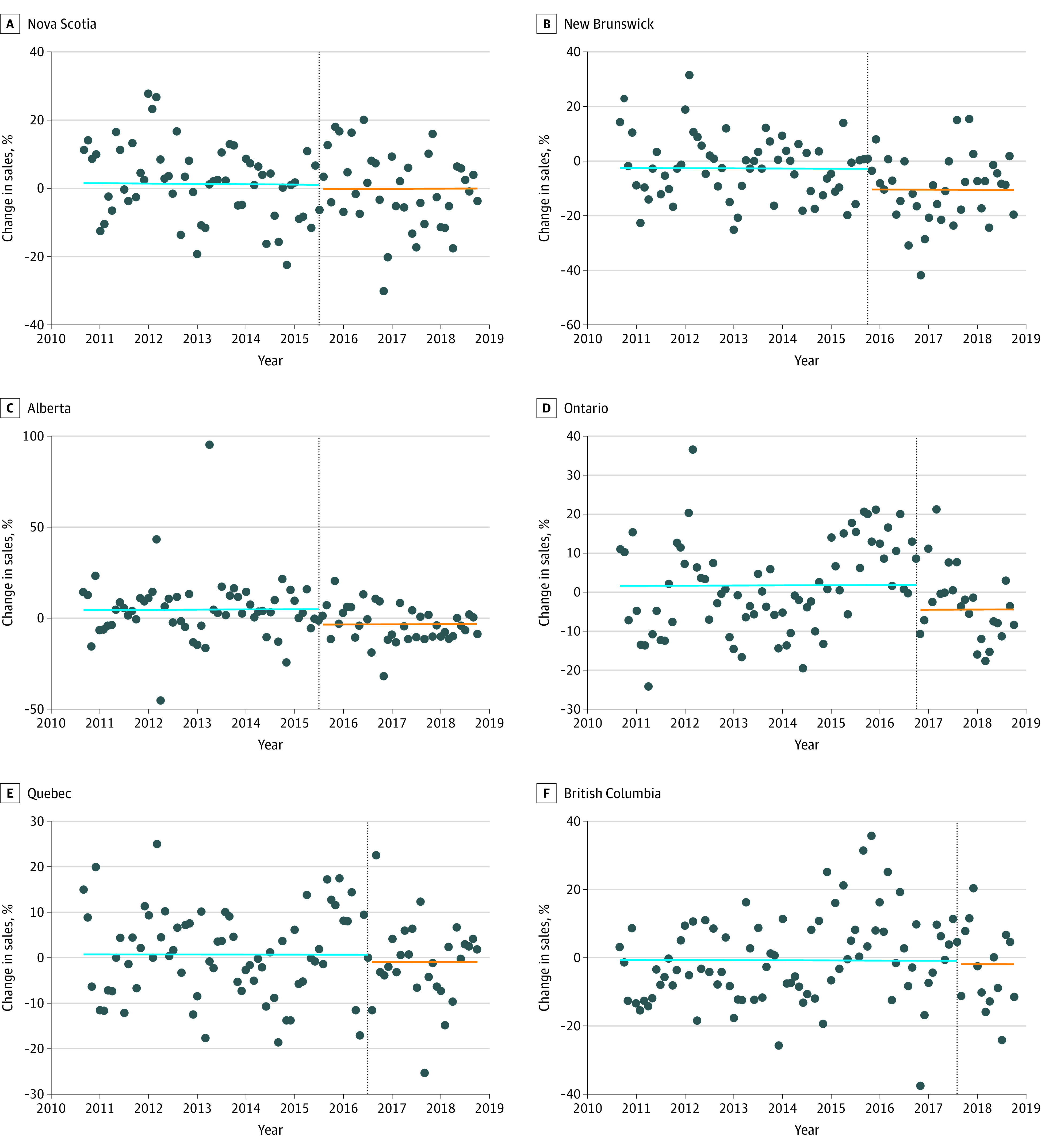
Trends in Overall Wholesale Cigarette Sales by Month in Canadian Provinces from 2010 to 2018 Each dot represents the current monthly sales in the identified province, with sales from the same month from the previous year subtracted to control for seasonality and to compare provinces on the same scale. The vertical lines indicate the implementation date of the menthol ban in that province. The blue lines represent the slope before the menthol cigarette ban, and the orange lines represent the change in slope after the ban.

In general, there was no significant trend in overall cigarette sales before implementation of the menthol cigarette bans (beginning in May 2015) (0.001%; 95% CI, –0.002% to 0.004%; *P* = .48). There was a nonsignificant decline in trend after the bans (−0.06%; 95% CI, −0.21% to 0.09%; *P* = .39) (Table). The postestimation test of the combined effect size of the ban on the magnitude (−4.6%; 95% CI, −8.2% to −1.0%) and trend (−0.06%; 95% CI, −0.21% to 0.09%) was significant (*P* = .02). The transformed Durbin-Watson statistic was 2.11, reflecting minimal residual autoregression.

## Discussion

This economic evaluation found that the bans restricting the sale of menthol cigarettes in Canadian provinces were associated with significant reductions in menthol cigarette sales and total cigarette sales. Previous behavioral studies have suggested that menthol cigarette smokers were likely to attempt to quit smoking after the ban.^4^ To our knowledge, this is the first study to evaluate the association between the menthol ban and overall cigarette sales by estimating the percentage change in cigarette sales. However, findings of the present study are consistent with those of previous research that have found associations between the ban and a decrease in sales of cigarettes in Ontario^4^ and a decrease in overall sales of menthol cigarettes in Canada.^6^ A limitation of the study is that some contraband cigarette sales were not included.
